# Inborn Errors of Metabolism Associated With Autism Spectrum Disorders: Approaches to Intervention

**DOI:** 10.3389/fnins.2021.673600

**Published:** 2021-05-28

**Authors:** Tamara Žigman, Danijela Petković Ramadža, Goran Šimić, Ivo Barić

**Affiliations:** ^1^Department of Paediatrics, University Hospital Center Zagreb and University of Zagreb School of Medicine, Zagreb, Croatia; ^2^Department of Neuroscience, Croatian Institute for Brain Research, University of Zagreb School of Medicine, Zagreb, Croatia

**Keywords:** autism spectrum disorder, early diagnosis, genetic testing, inborn errors of metabolism, therapeutic principles

## Abstract

Increasing evidence suggests that the autism spectrum disorder (ASD) may be associated with inborn errors of metabolism, such as disorders of amino acid metabolism and transport [phenylketonuria, homocystinuria, S-adenosylhomocysteine hydrolase deficiency, branched-chain α-keto acid dehydrogenase kinase deficiency, urea cycle disorders (UCD), Hartnup disease], organic acidurias (propionic aciduria, L-2 hydroxyglutaric aciduria), cholesterol biosynthesis defects (Smith-Lemli-Opitz syndrome), mitochondrial disorders (mitochondrial encephalomyopathy, lactic acidosis, and stroke-like episodes—MELAS syndrome), neurotransmitter disorders (succinic semialdehyde dehydrogenase deficiency), disorders of purine metabolism [adenylosuccinate lyase (ADSL) deficiency, Lesch-Nyhan syndrome], cerebral creatine deficiency syndromes (CCDSs), disorders of folate transport and metabolism (cerebral folate deficiency, methylenetetrahydrofolate reductase deficiency), lysosomal storage disorders [Sanfilippo syndrome, neuronal ceroid lipofuscinoses (NCL), Niemann-Pick disease type C], cerebrotendinous xanthomatosis (CTX), disorders of copper metabolism (Wilson disease), disorders of haem biosynthesis [acute intermittent porphyria (AIP)] and brain iron accumulation diseases. In this review, we briefly describe etiology, clinical presentation, and therapeutic principles, if they exist, for these conditions. Additionally, we suggest the primary and elective laboratory work-up for their successful early diagnosis.

## Introduction

Autism spectrum disorder (ASD) is a behavioral developmental disorder defined by the impairment of communication and social interaction. It usually starts before age three and children with autism can be recognized by stereotyped and repetitive patterns of behavior as well as their restricted activities and interests. ASD varies in degrees of severity, occurring in 1% of the population, and its prevalence has been increasing during the last three decades (Maenner et al., 2020). The causes of different subtypes of autism lay in the complex landscape of environmental, genetic and epigenetic influences (Waye and Cheng, 2018; Šimić et al., 2020). Although the etiology of most cases of ASD is still unknown, numerous studies have shown that there is a strong heritable component. Change (mutation or premutation) in the *FMR1* gene sequence on the chromosome X that results in fragile X syndrome (FXS) or fragile X-premutation tremor/ataxia syndrome (FXTAS) are among the leading genetic causes of ASD (Šarac et al., 2011; Ghaziuddin and Al-Owain, 2013). Current genetic testing strategies, including the combination of molecular cariotypisation and exome sequencing, are the most successful diagnostic approaches, yielding exact diagnosis in about 20–25% of the cases. The term “non-syndromic” autism refers to patients without dysmorphic characteristics or any other disease or additional signs or symptoms than those known to be associated with ASD. On the other hand, the term “syndromic” is being used to describe individuals with clinical characteristics additional to ASD, such as epilepsy, motor deficits, developmental delay and regression, dysmorphic features, and other manifestations or diseases that can contribute to autistic phenotype (Campistol et al., 2016).

It has been recently proposed that a significant number of ASD cases could be associated with various metabolic abnormalities, some of them identifiable only through untargeted metabolomic profiling, simultaneously opening additional space for therapeutic attempts (Glinton and Elsea, 2019).

### Inborn Errors of Metabolism

Clinical symptoms deriving from central nervous system (CNS) occur in more than 50% of patients with inborn errors of metabolism (IEM). In addition to neurological or somatic manifestations, psychiatric symptoms are also the presenting sign in 2−5% of IEM cases (Saudubray and García-Cazorla, 2018). Recent reports emphasized the causal role of IEMs in ASD, meaning that some IEM could be prevented, especially in population with high level of consanguinity (Celestino-Soper et al., 2012; Novarino et al., 2012). Many IEM are treatable conditions; in some of them positive effects are observed when treatments were started early in life. Phenylketonuria (PKU), the prototype of IEM successfully diagnosed by neonatal screening program and treated with diet if initiated early in life, is characterized by intellectual disability and autism (Asato et al., 2015). Possible IEM should be considered in the diagnostic approach of patients with syndromic ASD because for many of them there is a chance for intervention and improvement (Márquez-Caraveo et al., 2020). Spilioti et al. (2013) found significant proportion of patients with an IEM in patients with autism. For most of the IEM, ASD is not a single symptom, but accompanied with other neuropsychiatric and somatic disturbances (Simons et al., 2017). The data of Schiff and colleagues provide a large cohort of non-syndromic autistic subjects for whom a systematic metabolic work-up has been carried-out (Schiff et al., 2011). Their data suggest that usual metabolic workup should not be done routinely in non-syndromic ASD because the prevalence of IEM in the group of non-syndromic ASD is not higher than in general population (<0.5%). In the case of non-syndromic ASD, a precise clinical workup represents a good clinical practice (Schiff et al., 2011). In this review, we give an overview of IEM characterized by symptoms of ASD, propose diagnostic approach to assess such cases in clinical practice, and suggest possible specific therapies.

## ASD Associated With Inborn Errors of Metabolism

### Disorders of Amino Acid Metabolism

#### Phenylketonuria

Phenylketonuria (PKU) is the most common IEM in the group of aminoacidopathies. It is caused by biallelic mutations in the phenylalanine hydroxylase (PAH) gene with consequent lowering of PAH activity. PAH metabolizes phenylalanine to tyrosine, a process which requires the cofactor tetrahydrobiopterin (BH4). Deficient activity of PAH results in elevated concentration of phenylalanine in the blood and toxic levels in the CNS. If untreated, PKU is accompanied with severe and progressive intellectual disability, but may also be associated with symptoms such as autism, seizures, and motor deficits. Psychiatric or psychological difficulties may become apparent if the patient is not compliant with the diet (Blau et al., 2010). Newborn screening programs successfully diagnose patients with PKU. Treatment with a low phenylalanine diet in the postneonatal period has resulted in a worldwide population of over 50 thousand PKU individuals without cognitive deficits (ten Hoedt et al., 2011). BH4, the natural cofactor of PAH, may be effective in about 50% of PKU patients who are BH4-responsive to increase phenylalanine tolerance (van Wegberg et al., 2020). Possible new treatment options include enzyme therapy with phenylalanine ammonia lyase, enzyme that metabolizes phenylalanine, and gene therapy (van Wegberg et al., 2017).

#### Homocystinuria (Cystathionine β-Synthase Deficiency)

Cystathionine β-synthase (CBS) deficiency, also known as classical homocystinuria (HCU), is caused by biallelic mutations in the *CBS* gene. CBS deficiency prevents the conversion of homocysteine (Hcy) to cystathionine, resulting in homocysteine increase. Subjects with classical homocystinuria may have a clinical picture of varying severity and with different age of onset. It can start in childhood as a severe multisystemic disease or stay asymptomatic until adulthood (Morris et al., 2017). Untreated patients may have various symptoms, including osteoporosis, thromboembolic events, and intraocular lens dislocation. These patients can also have a variety of CNS disturbances, including symptoms of ASD (Abbott et al., 1987; Kiykim et al., 2016). Extrapyramidal signs and seizures are frequent features too (Morris et al., 2017). Treatment options include vitamins B_6_, B_12_ and folate supplementation, low-methionine diet and betaine (Kruger, 2017). Current efforts for developing novel therapies for HCU include enzyme replacement therapy with recombinant enzyme OT-58 that has been shown effective in lowering plasma and tissue homocysteine, ameliorating metabolic balance and clinical symptoms in a phase II clinical study (clinical trial NCT03406611). Gene therapy using adenovirus or minicircle DNA is also being appraised (Bublil and Majtan, 2020).

#### S-Adenosylhomocysteine Hydrolase Deficiency

S-adenosylhomocysteine hydrolase deficiency (SAHHD) is a rare disorder of methionine metabolism characterized by (not constant) hypermethioninemia, elevation of S-adenosylmethionine (AdoMet) and S-adenosylhomocysteine (AdoHcy, Barić et al., 2004). AdoHcy is an inhibitor of different methytransferases, enzymes that transfer methyl group to various molecules such as DNA, RNA, lipids, proteins, amino acids, and others. The removal of adenosine and Hcy under physiological conditions is sufficient to direct the flux toward hydrolysis. Hydrolysis of AdoHcy plays a critically important role in the regulation of reactions of biological methylation processes (Barić et al., 2004).

Clinical presentations vary from severe perinatal to milder forms and include various combinations of myopathy with elevated creatine kinase (hyperCKemia), behavioral disturbances, developmental delay, dysmyelination, coagulopathy, strabismus, and hepatic disease. Diet with low methionine intake can decrease and sometimes normalize plasma AdoMet and AdoHcy concentrations, with positive effects on methylation and clinico-biochemical parameters. Together with low protein/methionine diet, creatine and phosphatidylcholine supplementations have been used in some individuals, although without firm evidence of clinical improvement. Myopathy is less responsive to treatment, whereas liver, coagulation and neurological abnormalities are more responsive (Barić et al., 2017). Liver transplantation in a single patient improved cognitive development, especially gross motor, language and social skills (Strauss et al., 2015).

#### Branched-Chain α-Keto Acid Dehydrogenase Kinase Deficiency

Biallelic mutations in the *BCKDK* gene that codes for the kinase behind the negative regulation of the branched-chain α-keto acid dehydrogenase complex (BCKD) have been associated with clinical symptoms of autism with seizures (Novarino et al., 2012). Normalization of plasma branched-chain amino acids (BCAA) improves hyperactivity, attention span, gross motor and communication skills (Burrage et al., 2014). Supplementation with high-protein diet and frequent BCAA supplement dosing throughout the day are required for normalization of BCAA plasma concentrations (Burrage et al., 2014; García-Cazorla et al., 2014).

#### Urea Cycle Disorders

The urea cycle is a metabolic pathway that serves to eliminate excess of nitrogen, arising primarily as ammonia. Nitrogen in small quantities is essential substance for growth and cellular equilibrium, but excessive ammonia can lead to life-threatening consequences. In its non-ionized form ammonia easily crosses the blood-brain barrier and enters the CNS. In case of a severe metabolic crisis, a great amount of ammonia can accumulate in the blood and tissues, especially in the CNS that is most vulnerable. As the acute hyperammonaemia increases extracellular glutamate and causes excitotoxic cell death it comes as no surprise that neurological disturbancies such as ataxia, tremor, and seizures may ensue (Matsumoto et al., 2019). Urea cycle disorders (UCD) comprise deficiency of one of several enzymatic steps or transporters of the urea cycle. Clinical presentation is characterized by severe hyperammonaemic crisis in the newborn/infancy period, while the late-onset form is primary characterized by neurological symptoms from infancy through adulthood with possibility of metabolic decompensating and hyperammonaemia triggered by catabolic states. Besides other neurological symptoms, ASD was described in patients with carbamoyl phosphate synthetase deficiency, ornithine-transcarbamylase (OTC) deficiency, citrullinaemia type 1, and arginase deficiency (Frye, 2015). It is of immediate urgency to reduce the plasma ammonia concentration when hyperammonaemia develops. Acute management relies on ammonia detoxification by giving ammonia scavengers (sodium-benzoate or sodium-phenylbutirate), but hemodialysis and hemodiafiltration are the most efficient treatment strategies for plasma ammonia reduction. In parallel, catabolic state is reversed by providing high amounts of intravenous glucose and lipids. Arginine-hydrochloride is given to stimulate urea cycle reactions (except in case of arginase deficiency). Chronic treatment with low-protein diet prevents a catabolic state and recurrence of hyperammonaemia. In severe neonatal forms, prognosis is unfavorable if severe metabolic crisis develops. In late-onset forms improvement of symptoms has been reported with treatment. Liver transplantation can change the unfavorable clinical course in male patients with OTC deficiency (Matsumoto et al., 2019).

#### Hartnup Disease

Hartnup disease is a disfunction of the B^0^AT1 protein that results in overexcretion of neutral amino acids in feces and urine (Hashmi and Gupta, 2021). B^0^AT1 is a transporter responsible for the absorption of neutral amino acids in small intestine and renal tubules. As tryptophan serves as a precursor for niacin, deficiency of tryptophan and niacin manifests as pellagra-like skin changes and neurological disturbancies (Orbak et al., 2010). Along with ASD, clinical presentation includes reversible and intermittent episodes of neurological and symptoms affecting the skin. Neurological symptoms include ataxia, tremor, depression, mood disorders, epilepsy and psychosis. The skin eruptions are present at areas exposed to sun and have erythematous and scaly appearance. The disease is treatable with high protein diet and nicotinamide (Hashmi and Gupta, 2021).

Loss of CLTRN protein and its functions results in a disorder of similar biochemical phenotype to Hartnup disease. *CLTRN* gene encodes the protein collectrin, a homolog of angiotensin-converting enzyme 2 (ACE2), which is involved in transportation and activation of B^0^AT1 protein in the renal epithelium (Singer and Camargo, 2011; Pillai et al., 2019).

### Organic Acidurias

#### Propionic Aciduria

Propionic aciduria (PA) is an inborn error of branched chain amino acids metabolism, defined by accumulation of propionic acid due to deficiency of enzyme propionyl-CoA carboxylase (Baumgartner et al., 2014). Clinical features of PA may occur due to accumulation of toxic metabolites, altered mitochondrial energy metabolism, carnitine depletion, and coenzyme A sequestration. Acute illness may be presented by metabolic acidosis, acute alterations of consciousness or encephalopathy due to hyperammonaemia, anorexia, nausea, and vomiting. Chronic complications include poor growth, movement disorders, progressive spastic quadriparesis, epilepsy, cardiac dysfunction, osteopenia/osteoporosis, and functional immunodeficiency (Fraser and Venditti, 2016). Neurological complications include epilepsy, intellectual disability, and ASD (Ghaziuddin and Al-Owain, 2013). PA is treated with protein-restricted diet, precursor-free amino acid supplementation, and carnitine supplementation due to secondary carnitine deficiency. Acute treatment aims to stop catabolism and enable ammonia detoxification by giving sodium benzoate, L-arginine, and N-carbamyl-glutamate. Severe metabolic acidosis sometimes requires hemodiafiltration. Despite adequate therapy, significant number of patients have intellectual disability and psychiatric problems, thus requiring psychosocial support from childhood throughout adult life (Baumgartner et al., 2014).

#### L-2 Hydroxyglutaric Aciduria

L-2-hydroxyglutaric aciduria is a metabolic disorder affecting CNS and characterized by elevated concentrations of L-2-hydroxyglutaric acid in plasma, urine and cerebrospinal fluid (CSF) due to the deficiency of L-2-hydroxyglutarate dehydrogenase. Disease is defined by progressive neurological symptoms: ataxia, mental deterioration, subcortical leukoencephalopathy, and ASD (Zafeiriou et al., 2008). A few successful therapeutic trials with riboflavin and flavin adenine dinucleotide (FAD) have been reported in subjects with L-2-hydroxyglutaric aciduria. Yilmaz (2009) described the first patient treated with riboflavin in whom a partial improvement in motor and cognitive performance and significant decrease of urinary excretion of L-2-hydroxyglutarate was observed. Another approach, described by Samuraki et al. (2008) in an adult patient, is based on treatment with levocarnitine and FAD, which resulted in improvement of dystonia and tremor as well as in a significant decrease of urinary excretion of L-2-hydroxyglutarate. These examples suggest that supplementation of riboflavin improves the enzymatic activity by raising the intracellular concentration of FAD. Therefore, they may be categorized into a group of vitamin-responsive IEM (Van Schaftingen et al., 2009).

### Cholesterol Biosynthesis Defects

#### Smith-Lemli-Opitz Syndrome

Smith-Lemli-Opitz syndrome (SLOS) is an autosomal recessive disorder caused by biallelic mutations in the *DHCR7* gene, resulting in impaired biosynthesis of cholesterol. Besides dysmorphic features and intellectual disability, autistic symptoms are often a part of the SLOS behavioral phenotype (Thurm et al., 2016). SLOS is characterized by elevated 7-dehydrocholesterol and low plasma cholesterol levels. Some of the major consequences of cholesterol deficiency include abnormal formation of the CNS, face and limbs via an effect on sonic hedgehog (SHH) signaling pathways during development (Li et al., 2020) and its effect on steroidogenesis, particularly that of dihydrotestosterone. Therapeutic intervention with supplementation of cholesterol and symptomatic treatment after early diagnosis decreases mortality and improves long-term outcome (Donoghue et al., 2018). It is also important to know that several common antipsychotic, antidepressant, and anxiolytic compounds, including aripiprazole, buspirone, fluoxetine, haloperidol, nefazodone, perospirone, and trazodone may induce an *in vitro* biochemical profile similar to SLOS (Kim et al., 2016).

### Mitochondrial Disorders of Energy Production

Mitochondrial dysfunction is a common metabolic disturbance observed in ASD subjects. At the same time, different clinical features and biochemical abnormalities seen in ASD subjects can be linked to mitochondrial dysfunction (Frye and Rossignol, 2011). Although it seems that mitochondrial dysfunction can unify the seemingly disparate abnormalities associated with ASD, mitochondrial failure is commonly a secondary phenomenon related to other biological processes, such as chronic immune dysfunctions and increased oxidative stress that have been observed in ASD patients (James et al., 2009; Li et al., 2009; Malik et al., 2011).

Mitochondrial encephalomyopathy, lactic acidosis, and stroke-like episodes (MELAS) is one of the most frequent mitochondrial disorders, inherited maternally. Clinical criteria include: (1) stroke-like episodes before age of 40, (2) encephalopathy with seizures and/or cognitive deficits, and (3) mitochondrial myopathy accompanied by lactic acidosis and/or ragged-red fibers (El-Hattab et al., 2015). In addition to its neurological manifestations, MELAS syndrome can include a clinical picture of ASD (Ahmadabadi et al., 2020). Connolly and collaborators described kindred with MELAS, autism, cardiomyopathy, and rhabdomyolysis associated with the A3260G mtDNA mutation (Connolly et al., 2010). Treatment of MELAS syndrome with L-arginine, coenzyme Q, creatine monohydrate, and carnitine can be beneficial. Valproic acid, metformin, and dichloroacetate should be avoided (El-Hattab et al., 2015).

### Neurotransmitter Disorders

#### Succinic Semialdehyde Dehydrogenase Deficiency

Succinic semialdehyde dehydrogenase deficiency (SSADH-D) is a genetic disease caused by disrupted metabolism of the γ-amino butyric acid (GABA). Impaired activity of the mitochondrial enzyme SSAD leads to the accumulation of γ-hydroxybutyric acid (GHB). The SSADH-D has highly heterogeneous clinical picture, with varying degrees of autism, mental retardation, hypotonia, ataxia, epilepsy, and a delay in speech development. There has been no approved curative therapy for the disease yet, but vigabatrin can be beneficial by reducing accumulation of SSA and GHB through inhibition of GABA transaminase. Inhibition with vigabatrin, however, has no effect on peripheral GABA transaminase, so the GHB keeps rising and reaches the CNS (Pearl et al., 2016). Many therapeutic approaches are currently being studied (Didiášová et al., 2020).

### Disorders of Purine Metabolism

#### Adenylosuccinate Lyase Deficiency

Adenylosuccinate lyase (ADSL) deficiency is a disorder of purine metabolism that reduces purine *de novo* synthesis and purine nucleotide recycling. ADSL was first described in Jaeken and Van den Berghe (1984), who discovered succinylpurines in the plasma, urine, and CSF of a few patients with severe psychomotor delay and autism. Growth and development might be normal in the newborn period (Jurecka et al., 2015), but in severe forms neurological symptoms might be evident immediately after birth (Ciardo et al., 2001). They include acute and chronic encephalopathy, behavioral abnormalities, and ASD. Treatment relies on epilepsy treatment. Intervention with D-ribose administration, which increases levels of phosporibosylpyrophosphate and stimulates purine synthesis *de novo*, has only limited success (Jurecka et al., 2015).

#### Lesch-Nyhan Syndrome

Lesch-Nyhan syndrome (LNS) is an X-linked disorder caused by mutations in the *HPRT1* gene, coding for hypoxanthine-guanine phosphoribosyltransferase (HPRT). Symptoms include dystonia, intellectual disability, gout, autism, and behavioral changes such as self-mutilation. Pathogenesis is unclear and it remains to be uncovered how deficits in hypoxanthine and guanine recycling lead to a severe phenotype. Upon identification of several isoforms of amyloid precursor protein (*APP*) mRNA with a deletion followed by an insertion that accounted for epigenetic control of genomic rearrangements of *APP* gene in fibroblasts of patients with LNS, Nguyen proposed a role of epistasis between mutated *HPRT1* and *APP* genes affecting the regulation of alternative *APP* pre-mRNA splicing as a possible pathophysiological mechanism of the severe neurobehavioral phenotype of LNS (Nguyen, 2019). Patients excrete large amounts of uric acid. Allopurinol prevents urate nephropathy. Treatment with allopurinol has no effect on neurological symptoms (Bell et al., 2016).

### Cerebral Creatine Deficiency Syndromes

Creatine serves as a donor of high-energy phosphates for the synthesis of hydrolyzed adenosine triphosphate. The role of creatine transporter coded by the *SLC6A8* gene on chromosome X is to translocate creatine across the cell membrane in the cytoplasm of neurons and myocytes. Mutations in the *SLC6A8* gene lead to cerebral creatine deficiency syndrome (CCDS) with a spectrum of clinical manifestations, including severe intellectual delay, autism, epilepsy, and motor dysfunction. Females heterozygous for the *SLC6A8* pathogenic mutation are asymptomatic or have mild intellectual delay. The other two CCDS disorders are caused by the deficiencies of two enzymes involved in the synthesis of creatine: arginine-glycine amidinotransferase (AGAT) and guanidinoacetate methyltransferase (GAMT). All three conditions result in creatine depletion in the brain (Farr et al., 2020). GAMT and AGAT are treated with oral creatine monohydrate to restore cerebral creatine levels. Therapy of GAMT using ornithine supplementation and protein dietary restriction has only limited success (Mercimek-Andrews and Salomons, 2015).

### Disorders of Folate Transport and Metabolism

#### Cerebral Folate Deficiency

Cerebral folate deficiency (CFD) is any neurological condition with a low CSF concentration of 5-methyltetrahydrofolate (5-MTHF) and normal folate plasma concentrations. This syndrome is characterized by a wide variety of clinical symptoms, from irritability and sleep disturbances to severe seizures, developmental regression, autism, progressive ataxia and extrapyramidal symptoms, including choreoatethosis (Hyland et al., 2010). CFD is a treatable condition if timely recognition is followed by treatment with folinic acid perorally. Treatment with folic acid is contraindicated as it may exacerbate the 5-MTHF deficiency in the CNS. Autoimmunity against the folate receptor (transporter of 5-MTHF into the CSF) and mutations in the folate receptor 1 (FOLR1) gene are the primary causes of CFD. Secondary CFD has been observed in other IEM, including mitochondrial diseases, serine deficiency, and pyridoxine-dependent epilepsy (Pope et al., 2019).

#### Methylenetetrahydrofolate Reductase Deficiency

Methylenetetrahydrofolate reductase deficiency (MTHFR-D) is the most common genetic cause of hyperhomocysteinemia. MTHFR has an important role in the conversion of Hcy to methionine. A common genetic variant in the *MTHFR* gene is the c.677C > T polymorphism (rs1801133), where the common variant is less active at higher temperatures (Dean, 2012). In comparison to controls, subjects with two copies of this variant usually have higher Hcy levels and lower serum folate levels (“TT homozygous”). Another common *MTHFR* variant, c.1298A > C, is not associated with homocysteinemia alone, but only combined heterozygosity of c.1298A > C and c.677C > T results in an outcome similar to TT genotype. Pathogenic variants in the *MTHFR* gene can cause an autosomal recessive IEM with high concentrations of Hcy in plasma and urine. This, in turn, may cause developmental delay, thrombosis, eye disorders, and osteoporosis (Dean, 2012). Importantly, the TT genotype is frequent among ASD patients and their mothers, compared to the general population. The results of the study of Sadigurschi and Golan (2019) support the view that the maternal *MTHFR* genotype is associated with increased risk for ASD in children. The study of Orenbuch et al. (2019) showed that prenatal nutritional intervention by adding folic acid and choline in the diet of pregnant MTHFR-deficient mice reduce autistic-like behaviors among offspring that are MTHFR-deficient. Further studies are needed to better define the role of methionine cycle in the pathogenesis of ASD.

### Lysosomal Storage Disorders

#### Muccopolysaccharidosis Type III (Sanfilippo Syndrome)

Mucopolysaccharidosis type III (MPS III) is a multisystemic lysosomal storage disease characterized by progressive neurodegeneration characterized by severe developmental regression, intellectual disability, autism, and other behavioral problems. Multisystemic manifestations include hearing loss, musculoskeletal problems (contractures, joint stiffness, hip dysplasia and scoliosis), respiratory tract infections, and valvular cardiac disease (Wagner and Northrup, 2019). Patients are commonly misdiagnosed as having idiopathic developmental delay, attention deficit/hyperactivity disorder or ASD with unnecessary testing procedures and treatment attempts. Neurological decline is inevitable (Wijburg et al., 2013). Treatment is symptomatic with therapies such as enzyme replacement; however, the latest developments could push gene therapy into the mainstream (Seker Yilmaz et al., 2021).

#### Neuronal Ceroid Lipofuscinoses

Neuronal ceroid lipofuscinoses (NCL) are a group of autosomal recessive, progressive lysosomal storage disorders with dominantly neurological symptomatology. This group of diseases has a high genetic heterogeneity and variable clinical presentation. The most common forms are infantile NCL (INCL), late infantile NCL (LINCL), and juvenile NCL (JNCL). Common symptoms include epilepsy, developmental regression, and ophthalmologic abnormalities. Madaan et al. (2020) described a patient with autistic regression and epilepsy, along with EEG photosensitivity that is considered as an early sign of Batten disease. Cerliponase α, a recombinant human tripeptidyl peptidase 1 enzyme replacement therapy, became the first globally approved treatment for LINCL—Batten disease in 2017, signifying major therapeutic progress (Johnson et al., 2019).

#### Niemann-Pick Disease Type C

Niemann-Pick disease type C is an autosomal recessive disorder causedby mutations in *NPC1* (in 95% of cases) or *NPC2* (inabout 5% of cases) genes. When either of two proteins, NPC1 or NPC2, is not functional, it leads to impaired cellular trafficking of endocytosed LDL cholesterol and its accumulation in the lysosomes. The clinical presentation is highly heterogeneous, with an age of onset ranging from the perinatal period to late adulthood. Visceral signs such as fetal hydrops, hepatosplenomegaly, and cholestatic jaundice in early forms usually preceed neurological and psychiatric manifestations. Manifestations of neurological forms (late-infantile and juvenile forms) are ataxia, clumsiness, handwriting difficulties, impaired attention, vertical supranuclear gaze palsy and gelastic cataplexy. Some patients develop seizures. Compared to younger-onset patients, individuals with the adolescent/adult onset more frequently develop psychiatric symptoms and cognitive impairment (Newton et al., 2018).

Oxysterols (cholestane-3β, 5α and6β-triol and 7-ketocholesterol are elevated inmost patients, but they can also be elevated in the deficiency of lysosomal acid lipase and in neonatal cholestasis in general. Gene analysis is diagnostic. Miglustat is currently the only causal treatment for neurological manifestations (Geberhiwot et al., 2018; Patterson et al., 2020).

### Cerebrotendinous Xanthomatosis

Cerebrotendinous xanthomatosis (CTX) is a disorder of bile acid synthesis caused by mutations in the cytochrome P450 *CYP27A1* gene. These mutations cause dysfunction of sterol 27-hydroxylase enzyme, resulting in incomplete oxidation of the cholesterol side chain. Although CTX does not belong to the lysosomal diseases, by its clicial presentation it resembles storage diseases. Hence, it is often classified in this group. CTX is characterized by high levels of plasma cholestanol and accumulation of cholestanol and cholesterol in the CNS and tendons, which is manifested as tendon xanthomas. Typical clinical symptoms of CTX include chronic diarrhea, tendon xanthomas, bone fractures, bilateral cataracts, and neurological dysfunction. The disease is progressive, particularly due to severe neurological presentations that may include autism, intellectual disability, psychiatric and behavioral problems. Treatment with chenodeoxycholic acid is the current standard of care; if initiated early, it can stop the progression of the disease (Duell et al., 2018). At present, level of 7α-hydroxycholestenone appears to be the best biochemical marker for the evaluation of CTX treatment (Lütjohann et al., 2020).

Although Smith-Lemli-Opitz syndrome, Niemann-Pick disease type C and CTX all affect cholesterol metabolism and can present with psychiatric symptoms including autistic features, other clinical signs, metabolic background and treatment approaches do not overlap.

### Disorders of Copper Metabolism

#### Wilson Disease

The key clinical features of Wilson disease include acute episodes of hemolysis in association with acute liver failure, cirrhosis, neuropsychiatric disturbances including autistic features, and eye manifestations such as Kayser–Fleischer ring. Psychiatric and behavioral symptoms are common and may precede other neurological or hepatic signs and symptoms. Wilson disease is a disorder of copper metabolism, caused by biallelic mutations in the *ATP7B* gene, and characterized by low serum ceruloplasmin levels and elevated daily urinary copper excretion (Ferenci, 2017). Current treatment includes avoiding copper-rich foods, reducing copper absorption with zinc, chelation therapy to remove copper from tissues, and symptomatic treatment (Mulligan and Bronstein, 2020). Early recognition is prerequisite for treatment success (Mulligan and Bronstein, 2020).

### Disorders of Haem Biosynthesis

#### Acute Intermittent Porphyria

Mutations in hydroxymethylbilane synthase gene (*HMBS*) cause autosomal dominant acute intermittent porphyria (AIP). AIP is characterized by life-threatening neurovisceral attacks and increased risk of hepatocellular carcinoma, hypertension, and kidney failure. The only possible treatment is liver transplantation (Bustad et al., 2020). Luder et al. (2009) described a 15-year-old girl with AIP, whose first symptoms were autistic features at the age of 4. Visceral symptoms have occurred in the later course and the diagnosis of AIP was confirmed by the finding of a known causative AIP mutation (Luder et al., 2009).

A new therapeutic approach to AIP is RNA interference-based therapy (Givosiran), which decreases aminolevulinic acid syntase mRNA levels. Another promising approach is gene therapy that targets hepatocytes harboring mutated porphobilinogen deaminase (*PBGD*) gene (Spiritos et al., 2019).

### Brain Iron Accumulation Diseases

Neurodegeneration with brain iron accumulation (NBIA) is a group of genetic diseases characterized by movement disorders such as parkinsonism and dystonia, psychomotor delay, and early death. There is still no established therapy available to mitigate or stop the progression of these diseases (Di Meo and Tiranti, 2018). Two of these diseases are characterized by autistic regression as a dominant clinical symptom: panthotenate kinase-associated neurodegeneration (PKAN) and β-propeller protein-associated neurodegeneration (BPAN). Veeravigrom and colleagues described a girl diagnosed with ASD at the age of three, later confirmed to have PKAN. The brain MRI at 15 year of age showed abnormally low T2 signal intensity in the globus pallidus and posterior limb of internal capsule bilaterally due to iron accumulation (“eye of the tiger” sign), as well as cortical atrophy (Veeravigrom et al., 2014). Yogananthan and colleagues described a girl with seizures, autistic regression, intracranial calcification, and iron accumulation in the nucleus niger and globus pallidus. The diagnosis of BPAN was established by identification of pathogenic variant in WD repeat domain 45 (*WDR45*) gene encoding for β propeller protein. Current treatment is only palliative (Yoganathan et al., 2016).

## Diagnostic and Therapeutical Approaches

Many IEM present with syndromic ASD, most commonly as an associated symptom. Diagnostic approach includes wide neuroradiologic, metabolic, and genetic workup (Polšek et al., 2011). In this review, we briefly describe etiology, clinical presentation, and therapeutic principles, if available, for several groups of IEM in differential diagnosis of ASD. Many of them are nowadays diagnosed and successfully prevented by neonatal screening program. Based on the list of potentially treatable IEM mentioned in this paper, we suggest following laboratory work-up: ammonia and lactate concentration, acid-base balance, plasma amino acid analysis, urinary organic acid analysis, urinary amino acids, plasma or dry blood spot acylcarnitine profile, total cholesterol, total homocysteine, vitamin B_12_ concentration, and uric acid. Further, more specific laboratory tests such as purine and pyrimidine analysis, CSF analysis (amino acids, folic acid concentration), cholestanol concentration, urinary glycosaminoglycans and porphyrins, copper excretion, and ceruloplasmin concentration should be done based on additional leading symptoms or laboratory results, but should not be a part of the primary workup. All laboratory tests should be done after a detailed anamnesis and clinical examination with special attention on associated neurological abnormalities. Brain MRI with spectroscopy and electroencephalogram have their place in the primary workup of an ASD patient due to several specific findings that can be leading to a proper diagnosis (CCDSs, NBIA). Here, we do not focus on common genetic causes of ASD such as FXS, tuberous sclerosis, Rett syndrome or on rare dysmorphic syndromes and genetic diagnostic tests such as molecular karyotyping (copy number variants could be found in 10−15% of cases), PCR assay or single specific gene analyses. In the end, unspecific genomic approaches like whole exome sequencing could be useful in diagnosing rare disorders missed by proposed diagnostic workup and will reveal the etiology in about 30−40% of syndromic ASD cases (Schaefer et al., 2013).

Over the years, ample evidence has accumulated to suggest that children with ASD have different biochemical profiles compared to healthy children. For example, age-independent hyperserotonemia is present in approximately one-third to one-half of subjects with ASD (Šimić et al., 2020). Untargeted metabolomics, although still not implemented in routine clinical practice, has been shown to be a promising approach in screening of underlying biochemical abnormalities, discovering new specific biomarkers and directing treatment (Glinton and Elsea, 2019).

An interesting study was done in Greece where a large cohort of children with confirmed ASD were screened for the presence of IEM (Spilioti et al., 2013). Their data provide the evidence for a new potential biomarker—3-hydroxyisovaleric acid as well as a few novel treatment approaches for children with ASD: biotin supplementation and a ketogenic diet elicited mild to significant improvement in clinical picture (Spilioti et al., 2013).

Basic six therapeutic principles of IEM include: (1) substrate reduction therapy (as in PKU), (2) removal of toxic metabolites (e.g., urea cycle disorders), (3) product supplementation (e.g., cerebral creatine deficiency disorders), (4) stimulating of deficient enzyme activity (e.g., B_6_ supplementation in CBS deficiency), (5) enzyme replacement therapy (e.g., in LINCL) or 6) organ transplantation (in some cases of UCD and AIP). As exemplified in parentheses, these principles can be also observed throughout the IEM listed above.

### Methionine Cycle and its Disturbances as Well as Folic Acid Enigma Deserve Special Attention Regarding Therapeutic Trials and Opinions

Periconceptional folic acid supplementation in pregnant women along with the dietary and blood folate concentrations in children with ASD have been shown as environmental factors contributing to the incidence of autism. Moreover, children with autism have hyperhomocysteinemia compared to controls, whereas serum levels of folate and vitamin B_12_ may be diminished or normal. This can be due to lower folate intake in these subjects. Furthermore, folinic acid supplementation in ASD subjects with low 5-MTHF levels in CSF leads to the normalization of folate levels and alleviates some symptoms of ASD (Castro et al., 2016).

There are several examples of enzyme deficiencies in methionine cycle (CBS, SAHHD, MTHFR) with clinical presentation of ASD. It is yet to be elucidated how subtle disturbances in this metabolic pathway affect methylation and consequent epigenetic dysregulation of numerous genes.

Malaguarnera and Cauli (2019) showed the positive clinical effects of L-carnitine administration in non-syndromic forms of autism. Several clinical trials suggest that carnitine supplementation is useful in diminishing symptoms of non-syndromic ASD. Nevertheless, future clinical trials to identify the subgroup of ASD patients that could benefit from carnitine supplementation are needed (Malaguarnera and Cauli, 2019).

Treating an autistic child is always associated with additional healthcare-associated costs, therapies, education, and family services. The associated social and emotional stresses often lead to depression, somatization, and impairment in quality of life parameters (Glinton and Elsea, 2019).

**FIGURE 1:**
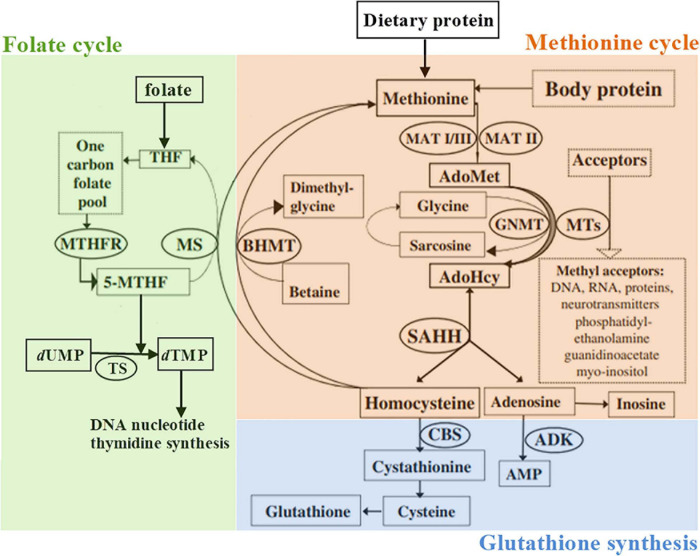
Schematic representation of the methionine metabolism according to Barić et al. (2017), slightly modified. 5-MTHF, 5-methyltetrahydrofolate; ADK, adenosine kinase; AdoHcy, S-adenosylhomocysteine; AdoMet, S-adenosylmethionine; AMP, adenosine monophosphate; BHMT, betaine-homocysteine methyltransferase; CBS, cystathionine β-synthase; GNMT, glycine *N*-methyltransferase; dTMP, 2′-deoxythymidine-5′-monophosphate (thymidylate); dUMP, 2′-deoxyuridine-5′-monophosphate; MAT, methionine adenosyltransferase; MS, methionine synthase; MTHFR, methylenetetrahydrofolate reductase; MTs, a variety of AdoMet-dependent methyltransferases; THF, tetrahydrofolate; SAHH, S-adenosylhomocysteine hydrolase; TS, thymidylate synthase.

At the present time, there is not enough evidence for routine supplementation of ASD patients with folic acid, choline, or other supplements. We suggest methionine cycle (Figure 1) to be carefully analyzed in syndromic ASD patients (as proposed earlier, this should be performed by plasma amino acid analysis and total Hcy concentration measurement) and specific supplementation given in case of biochemically proven deficiencies.

## Conclusion

Symptoms of ASD can be a present in many IEM, but rarely occur in isolation. Since some of IEM are treatable, physicians treating children with ASD should be aware of a long list of rare and ultra-rare disorders in the differential diagnosis. Given the enormous etiologic variability of autistic symptoms, including those of genetic and metabolic origin, diagnostic work-up of these patients should include appropriate genetic and metabolic studies elected on the basis of leading symptoms and associated clinical signs. This review is also intended to serve as a reminder on IEM that could present with ASD as a leading or associated presentation. As always, detailed medical history and clinical examination, including detailed neurological examination, should be a basis for planning focused diagnostic work-up of patients with ASD.

## Author Contributions

IB conceived the manuscript. TŽ wrote the manuscript. All authors critically revised the manuscript and approved the final version.

## Conflict of Interest

The authors declare that the research was conducted in the absence of any commercial or financial relationships that could be construed as a potential conflict of interest.
